# Comparison and convergent validity of five Mediterranean dietary indexes applied to Brazilian adults and older adults: data from a population-based study (2015 ISA-Nutrition): a critical analysis

**DOI:** 10.1017/jns.2023.59

**Published:** 2023-07-17

**Authors:** Jane Cho, Vrushank Shah, David Lo

**Affiliations:** 1 American Preventive Screening & Education Association (APSEA), Stratford, NJ, USA; 2 Department of Biology, Rutgers, The State University of New Jersey, New Brunswick, NJ, USA; 3 Department of Medicine, Rowan University School of Osteopathic Medicine, Stratford, NJ, USA; 4 Department of Medicine, Rutgers Robert Wood Johnson Medical School, New Brunswick, NJ, USA

We read with pleasure the article by Bastos *et al.*^(1)^, titled ‘*Comparison and convergent validity of five Mediterranean dietary indexes applied to Brazilian adults and older adults: data from a population-based study (2015 ISA-Nutrition)*’. We would like to offer additional commentary on the extrapolated conclusions, particularly generalisability of research findings to global populations, impact of pre-existing disorders, socioeconomic status and the COVID-19 pandemic that could impair access to food and adherence to the Mediterranean diet. We hope these perspectives may provide insight and engender further research and development.

First, the study did not address adherence to the Mediterranean diet in individuals from other countries and focused primarily on Brazil. A study from the InCHIANTI cohort study of individuals in Italy showed that greater adherence to the Mediterranean diet resulted in reduced mortality. The study evaluated dietary habits of older adults of 65 and older with food frequency questionnaires developed for the Italian population^(2)^. In addition, a study conducted on Portuguese adults showed that there was a higher adherence to the Mediterranean diet if individuals were female, ate a large number of meals in a single day, and partook in activities in nature^(3)^. This study showed that different parts of the world may be impacted by distinct factors and it may be difficult to generalise the findings of the current study to populations across the globe. As a result, further data acquisition and research is necessary from multiple regions of the world and draw reliable conclusions on adherence to the Mediterranean diet at a global level.

Second, the study excluded individuals with chronic alcoholism, however, they did not rule out individuals with substance abuse disorders, eating disorders and mental health conditions that would affect one's lifestyle. A substance abuse survey in Brazil led to findings that substance abuse was highly prevalent in the region^(4)^. The survey claimed that substance abuse disorders decreased food consumption and nutrient absorption, which interfered with satiety regulation and nutrition^(5)^. This provides context for substance abuse to be included in the exclusion criteria of the study, as it impedes proper dietary habits in individuals and individuals with substance abuse disorder may find it difficult to adhere to the Mediterranean diet. In addition, other conditions, such as anxiety, depression and obesity, were found to influence food addiction, based on a study conducted on young adults at a Brazilian University^(6)^. Due to mental health conditions, binge eating and food consumption increased to compensate for the individual's respective condition. At the same time, eating disorders, such as bulimia nervosa, anorexia nervosa and atypical eating disorders should also be excluded from the study, as they can impact the amount of food intake and skew results measuring the adherence to the Mediterranean diet. If individuals eat too much or too little based on their eating disorder, then that may impact the validity and reliability of the results. A stronger exclusion criterion is necessary to verify the accuracy of the results in the study.

Third, socioeconomic status of individuals and social determinants to food access may impact dietary choices and impact adherence to the Mediterranean diet. Studies show that socioeconomic status impacts adherence to the Mediterranean diet and dietary costs associated with this diet. This implies that individuals who are of relatively lower socioeconomic status may see dietary costs as a larger barrier to adhering to the Mediterranean diet^(7)^. Moreover, the study did not account for barriers to food access. A 2019 study found that a greater distance to purchase groceries was associated with food insecurity in elderly populations, which may have restricted their ability to acquire certain foods like fruits or vegetables^(8)^. This suggests that the variable of the difficulty attaining groceries, such as legumes and lentils could hinder adherence to the Mediterranean diet. It indicates the need for a common index for socioeconomic status as well as appropriate measures to combat barriers to food access, ensuring proper adherence of research participants.

In addition, as the study was conducted in 2015, the recent effects of the COVID-19 pandemic may have caused disruptions in eating habits and dietary patterns, which change the current landscape of the article's conclusions. Specifically, the pandemic negatively impacted the measure of health-related quality of life, or an individual's or group's perceived perspective of their health, which provides insight into one's well-being. This could present an obstacle to following a healthier diet such as the Mediterranean diet^(9)^. Moreover, some studies found that the COVID-19 pandemic increased meal frequency and increased the consumption of snacks and unhealthy foods, however, other studies have found that consumption of fresh produce and increases in alcohol consumption were noted^(10)^. These conclusions indicate that future research that includes post-pandemic data would be necessary to expand upon the article's claims and gain greater insight into eating habits and adherence to specific diets.

In the end, we applaud the authors for synthesising a complex and highly involved study. We look forward to reading about future studies that provide insight into the factors discussed above.
